# Investigation of Head Kinematics and Brain Strain Response During Soccer Heading Using a Custom-Fit Instrumented Mouthguard

**DOI:** 10.1007/s10439-023-03430-8

**Published:** 2024-01-19

**Authors:** M. Barnes-Wood, H. McCloskey, S. Connelly, M. D. Gilchrist, A. Ni Annaidh, P. S. Theobald

**Affiliations:** 1 Cardiff School of Engineering, Cardiff University, The Parade, Cardiff, CF24 3AA UK; 2 Charles Owen & Co, Croesfoel Industrial Park, Wrexham, LL14 4BJ UK; 3 Football Association of Wales (FIFA Medical Centre of Excellence), Hensol, Pontyclun, CF72 8JY UK; 4 School of Mechanical and Materials Engineering, University College Dublin, Belfield, Dublin 4, Ireland

**Keywords:** Head kinematics, Finite element model, Head impact sensor, Strain response, Sub-concussive head impacts

## Abstract

Association football, also known as soccer in some regions, is unique in encouraging its participants to intentionally use their head to gain a competitive advantage, including scoring a goal. Repetitive head impacts are now being increasingly linked to an inflated risk of developing long-term neurodegenerative disease. This study investigated the effect of heading passes from different distances, using head acceleration data and finite element modelling to estimate brain injury risk. Seven university-level participants wore a custom-fitted instrumented mouthguard to capture linear and angular acceleration-time data. They performed 10 headers within a laboratory environment, from a combination of short, medium, and long passes. Kinematic data was then used to calculate peak linear acceleration, peak angular velocity, and peak angular acceleration as well as two brain injury metrics: head injury criterion and rotational injury criterion. Six degrees of freedom acceleration-time data were also inputted into a widely accepted finite element brain model to estimate strain-response using mean peak strain and cumulative strain damage measure values. Five headers were considered to have a 25% concussion risk. Mean peak linear acceleration equalled 26 ± 7.9 g, mean peak angular velocity 7.20 ± 2.18 rad/s, mean peak angular acceleration 1730 ± 611 rad/s^2^, and 95th percentile mean peak strain 0.0962 ± 0.252. Some of these data were similar to brain injury metrics reported from American football, which supports the need for further investigation into soccer heading.

**Supplementary Information:**

The online version contains supplementary material available at 10.1007/s10439-023-03430-8.

## Introduction

Association football, known as soccer in some regions, is the most popular global sport. Participant numbers were estimated at 265 M in 2007, including over 13 M women players [1]. Participation has several benefits including improved cardiovascular and mental health, and well-being [2, 3]. Recently, potential risks associated with the sport have gained attention, particularly in relation to head injuries [4]. Of specific concern is repeated ball heading [5]. This occurs during both gameplay and training, where players intentionally strike the ball with their head to gain a competitive advantage, including goal-scoring. Recent interest in sports-related repetitive head impacts, such as soccer heading, have motivated researchers to better understand the risks of mild traumatic brain injuries (mTBI) including concussion, and how these may increase the risk of longer-term neurodegeneration. Football Associations including in England and Wales have recently implemented guidance focused on a maximum 10 high force practice headers per week, for better player welfare [6].

Chronic traumatic encephalopathy (CTE), or dementia pugilistica [7], is a neurodegenerative condition identifiable during post-mortem examination, which is believed to be caused by repeated head impacts [8]. Defined by specific changes in neurofibrillary tangles and hyperphosphorylated tau protein deposition [9], risk of developing CTE is now known to increase for those who participate in elite, male American football [10]. Studies considering elite-level male soccer players, typically playing in the 1960’s and 70’s, have identified a higher mortality rate attributed to neurodegenerative disease, than in an age-matched control group [11, 12]. Position-specific data indicates heading exposure may be a potential cause, with a 5- and 3.5- fold greater CTE risk for defensive and attacking players, respectively, versus goalkeepers [13]; however, confounding factors (e.g. genetics, lifestyle, etc) and the lag between exposure and post-mortem examination, prevents a conclusive correlation.

Recent studies have focused on prospectively correlating sporting activity with head/brain kinematics (e.g. linear, rotational acceleration) [14–20], with some focussing on soccer heading [21, 22]. Equipment- or skin-mounted sensors have enabled data collection across a wide variety of sports, including soccer [23–26]. Such technologies are typically wireless and need to be very small, meaning acquisition can be prone to issues including inaccuracies and false positives [27]. Custom-fit instrumented mouthguards (iMGs), where the sensor is embedded within the polymer which, in turn, is fitted to the upper dentation of the athlete and so forming a direct coupling to the skull, have been shown to more accurately capture head kinematics [14, 28]. Investigators have then used these measures to explore relative brain injury risk, including via finite element (FE) analyses. Such an approach also aids understanding sub-concussive impacts, which lack clinical sequelae.

This study aims to understand the brain injury risk associated with soccer heading, adopting iMGs to collect kinematic data from a controlled population of amateur players. FE analysis is then used to estimate the brain strain associated with each heading event, before kinematic and strain-based injury metrics are applied to understand injury risk.

## Materials and Methods

### Participants

Seven male amateur soccer players (age = 20.1 years ± 1 year; height = 178.6 cm ± 5.9 cm; weight = 69.4 kg ± 7.6 kg) were recruited from a university soccer league. Participants provided written, informed consent following approval of the study by our Institutional Review Board.

### Experimental Setup

**Fig. 1:**
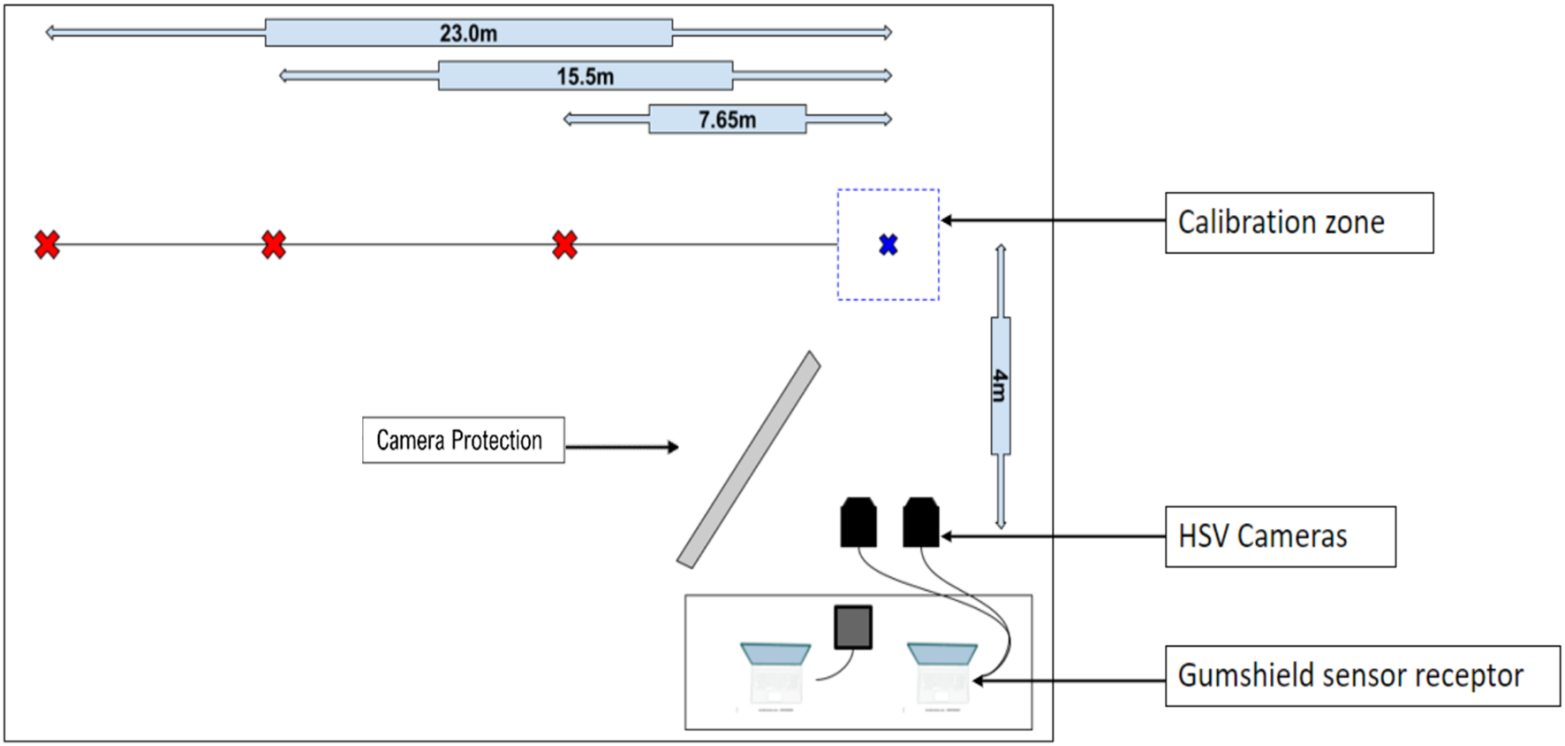
Experimental setup detailing three pass distances (red crosses), relative to the participant (blue cross).

A laboratory methodology was established to replicate three scenarios that commonly lead to a header: a throw in (hereafter termed a “short pass”); a corner kick (“medium pass”), and a long, lofted kick (“long pass”) (Fig. 1). Consistent ball delivery was achieved using a Globus EuroGoal 3000, launching an Adidas Champions League 21/22 soccer ball inflated to 0.8 bar (mass = 434.17 grams ± 0.35 grams), which is within the FIFA Quality Pro regulations [29]. Each participant headed 3 short, 3 medium and 4 long passes, spaced evenly across the 60-minute test period, which was consistent with the Football Association guidelines [6]. Ball velocities were approximately 8 ms^−1^, 13 ms^−1^, and 16 ms^−1^ for short, medium, and long pass distance, respectively. Participants were instructed to return the ball back towards the delivery location, using the frontal portion of their head, only attempting to head those deliveries where they could achieve this objective. All impacts were video recorded, using 2 cameras positioned on tripods, one at a perpendicular distance of 4 m from the impact location, the second angled 7° and 0.5 m from camera 1, capturing a binocular view. Both cameras [Mini AX200, Photron Europe; resolution: 1024 × 672 pixels; sample rate: 10000fps] began recording simultaneously when triggered, capturing high-speed video to validate true positive events by cross-referencing time-stamped data against iMG data.

### Head Impact Data Collection

Kinematic data were recorded during each header using an iMG (Protecht; Sports and Well Being Analytics, Swansea, UK.), designed for measuring impacts in non-helmeted, collision sports. These iMGs have previously been validated for impacts lower than 70 g, to provide kinematic measurements that allow for accurate calculation of brain injury metrics [30]. Dental impressions were taken prior to data collection, allowing for custom manufacture of each iMG device against each participant’s upper dentation (Opro; Hertfordshire, UK). The iMG main board comprised: a 3-axis accelerometer (H3LIS331DL; STMicroelectronics, Genova, Switzerland), sampling at 1 kHz (± 200 g, 16-bit resolution); a 3-axis gyroscope (LSM9DS1; STMicroelectronics, Genova, Switzerland)) sampling at 0.952 kHz (± 35 rad s^−1^, 16-bit resolution); and an additional accelerometer with a focussed bandwidth (0.5–1 kHz) [28]. This iMG configuration is consistent with previous studies [14–16], with an on-board proximity sensor ensured properly siting to the upper dentation. The iMG was programmed to trigger data collection when a threshold of 5 g was met in any of the X, Y or Z directions for linear acceleration. A 5 g threshold was selected over the usual 10 g threshold, as the controlled nature of the heading events allowed for accurate distinction between false-positive and true positive events. Once the threshold was achieved, a 104 ms window of linear and angular acceleration data was collected. This data was wirelessly transmitted to a receiver unit, connected to a secure cloud-based storage and analysis system.

### Kinematic Data Processing

All iMG data was manually cross-checked against high-speed video, to achieve visual confirmation of positive header events. Neither linear, rotational acceleration, nor rotational velocity data was filtered, as a Fast Fourier Transform did not identify any high-frequency components. Peak linear acceleration (PLA), peak angular acceleration (PAA) and peak angular velocity (PAV) were then identified for each header. The orientation and angular offset of the mouthguard sensors were determined using MRI data, captured on a 3 T Siemens Connectom (300mT/m). Further details can be found in the supplementary information. Raw kinematic data was estimated at the centre of gravity (CoG) of the head using multiple transformations, also detailed in the supplementary information. This enabled extraction of X, *Y*, *Z* linear and *X*, *Y*, *Z* angular acceleration-time data, for input into FE modelling.

### FE Modelling

 The University College Dublin Brain Trauma Model (UCDBTM) [31, 32] was adopted to simulate brain strain during each header. The head geometry was previously developed using imaging datasets of adult male cadavers and is formed of the scalp, skull, pia, falx, tentorium, cerebrospinal fluid, grey and white matter, cerebellum, and brain stem. The model contains approximately 26,000 hexahedral elements and comprises material property data as per Tables 1 and 2 [33–37]. The shear characteristics of the viscoelastic brain tissue have previously been defined for the model defined using Eq. 1.

**Table 1:** UCDBTM material properties

Material	Young’s modulus (MPa)	Poisson’s ratio	Density (kg/m^3^)
Scalp	16.7	0.42	1000
Cortical bone	15,000	0.22	2000
Trabecular bone	1000	0.24	1300
Dura	31.5	0.45	1130
Pia	11.5	0.45	1130
Falx and tentorium	31.5	0.45	1130
CSF	15,000	0.5	1000
Gray matter	Hyperelastic	0.49	1060
White matter	Hyperelastic	0.49	1060

**Table 2.** Viscoelastic UCDBTM material properties

Material	*G*_0_	*G*_∞_	Decay constant (s^-1^)	Bulk modulus (GPa)
Grey matter	10	2	80	2.19
White matter	22.5	4.5	80	2.19
Brain stem	12.5	2.5	80	2.19
Cerebellum	10	2	80	2.19

1 $$G\left(t\right)={G}_{\infty }+({G}_{0}-{G}_{\infty }){e}^{-\beta t}$$

where *G*_∞_ is defined as the long-term shear modulus, *G*_0_ is the short-term shear modulus and *β* is the decay factor.

Model validation was achieved by comparison of real-world brain injury events and cadaveric impacts performed by Nahum et al. [38–42], whilst brain motion data was captured by Hardy et al. to validate the shear deformation behaviour [41]. Gilchrist and Horgan reported good correlation between their model and experimental tests. A mesh refinement study was undertaken by Horgan and Gilchrist to ensure better agreeability between the model and the model outputs [31, 32]. Post et al. [43] reported the UCDBTM to generate large strain deformation data when only linear acceleration curves were used during loading. To ensure confidence in the use of this model for this study, a separate analysis was performed on a randomly selected simulation set, which can be found in Supplementary Information 2.

The transformed linear and angular acceleration time-series were applied to the CoG as a boundary condition, to determine the estimated deformation of the brain tissue during each header. Analysis was restricted to the cerebrum, in line with previous FE studies investigating influence of head kinematics on brain strain [44–49]. Simulations were performed in commercially available FE modelling software (Abaqus; Dassault Systemes), using a double precision explicit solver.

### Head injury Metrics

Kinematic values were used to calculate various brain injury criteria that predict head injury risk. Linear acceleration, angular acceleration, and angular velocity were analysed for kinetic definition. Common brain injury metrics were calculated to quantify brain injury risk: head injury criterion (HIC) and rotational injury criterion (RIC). HIC is considered the most widely used metric to measure the likelihood of an impact causing a head injury [50]. It considers linear acceleration only and is calculated using Eq. 2:

2 $${\text{HIC}}= {\left[\left({t}_{2}-{t}_{1}\right){\left\{\frac{1}{{(t}_{2}-{t}_{1})}{\int }_{{t}_{1}}^{{t}_{2}}a\left(t\right)\mathrm{ d}t\right\}}^{2.5}\right]}_{{\text{max}}}$$

where *a*(*t*) is resultant linear acceleration [*g*]. Time points *t*_2_ and *t*_1_ [*s*] report the maximised value of HIC and do not exceed a time interval of 0.015 s.

RIC utilises rotational acceleration [51] and is defined as:

3 $${\text{RIC}}= {\left[\left({t}_{2}-{t}_{1}\right){\left\{\frac{1}{{(t}_{2}-{t}_{1})}{\int }_{{t}_{1}}^{{t}_{2}}a\left(t\right)\mathrm{ d}t\right\}}^{2.5}\right]}_{{\text{max}}}$$

where *a*(*t*) is resultant angular acceleration [rad/s^2^]. Time points *t*_2_ and *t*_1_ [*s*] report the maximised value of RIC and do not exceed a time interval of 0.036 s.

### Evaluating Brain Strain Response

Two strain metrics were used to quantify the brain response during each heading impact: maximum principal strain (MPS) and CSDM. To report the severity of brain tissue deformation more accurately, ninety-fifth percentile MPS values were chosen to ensure mitigation of false strain values encountered in single elements. The MPS values extracted from the FE models were classified into established categories based on brain injury risk: very low (0*–*0.079 strain), low (0.08–0.169 strain), medium (0.17–0.259 strain), high (0.26–0.349 strain), and very high (> 0.35 strain) [17]. The medium category has been defined using biomechanical white and grey matter data and describes a 50% risk of concussion [17]. CSDM reports the cumulative volume fraction for every element exceeding a MPS threshold [52]. In this study, a 10% threshold is adopted (CSDM10), presenting the volume fraction of elements exceeding MPS 0.1 [52].

## Results

Seventy headers were performed, though only 65 iMG events were acquired due to data recording challenges. No clinical concussions or other symptomatic head injuries were reported during experimental testing.

### Brain Kinematic Data Analysis

**Fig. 2:**
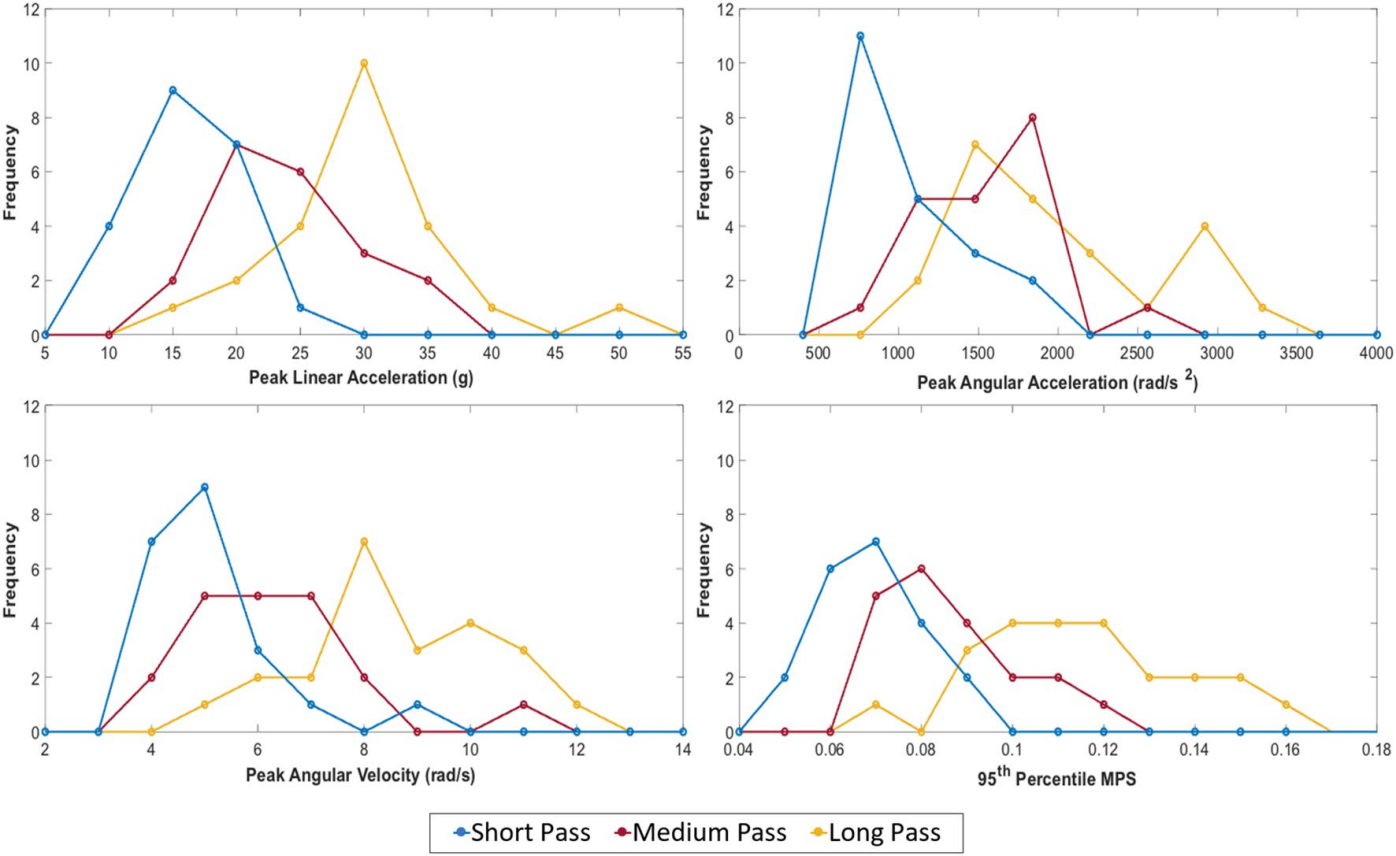
Frequency distributions of heading impacts (*n* = 65) detailing peak head kinematics and 95th percentile MPS across three pass distances

The resultant traces (linear and angular acceleration, and angular velocity, against time) were analysed, identifying the peak values. Fig. 2 shows the frequency distributions of PLA, PAA, PAV and MPS across all 65 header events, showing a skew towards the lower values. PLA values ranged from 14.2 to 52.1 g, with a median value of 24.3 g and a mean (± SD) of 26 g (± 7.9 g). PAA values ranged from 816.4 to 3391.7 rad/s^2^ with a median value of 1597 rad/s^2^ and a mean (± SD) of 1730 rad/s^2^ (± 611 rad/s^2^). Further, PAV values ranged from 4.12 to 12.1 rad/s, with a median value of 6.75 rad/s and a mean (± SD) of 7.20 rad/s (± 2.18 rad/s). MPS had a range of 0.0562 to 0.1646, with a median of 0.0927 and a mean (± SD) of 0.0962 (± 0.252).

**Fig. 3:**
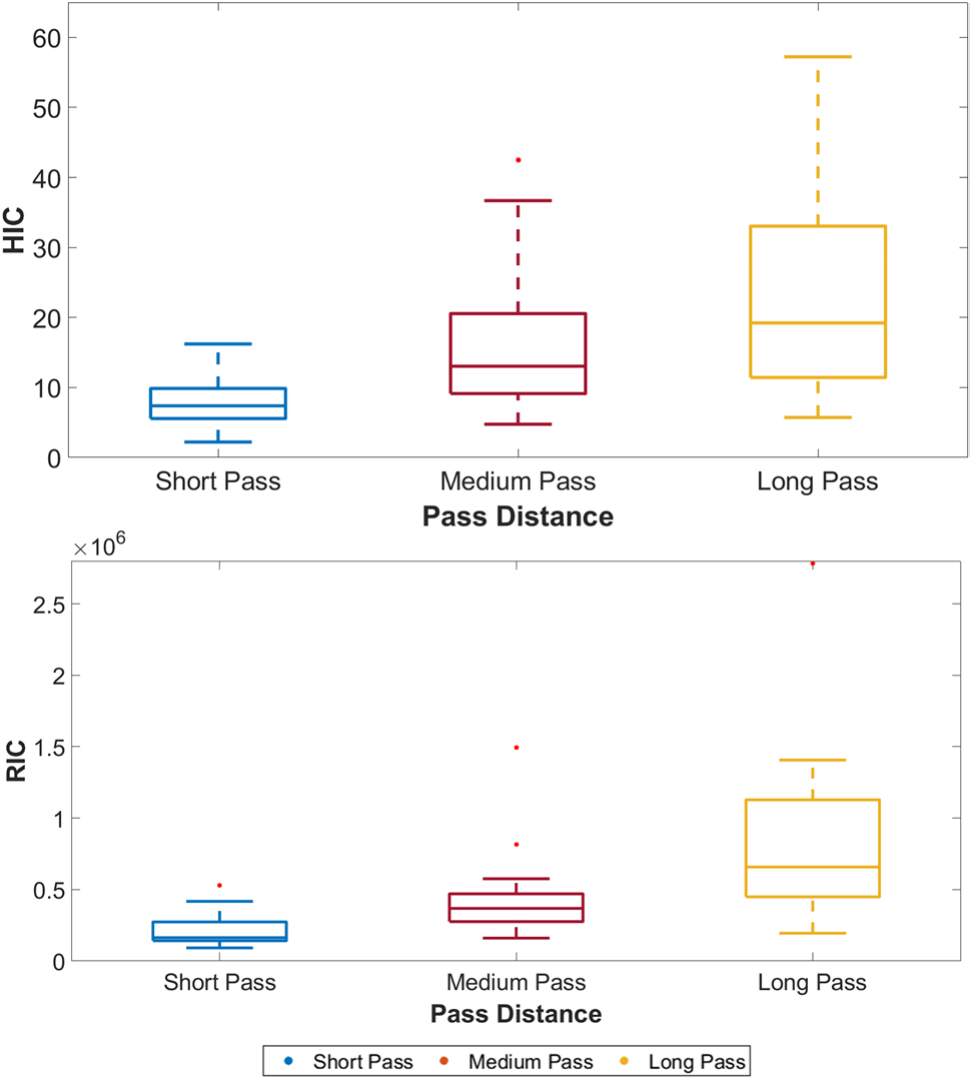
Box and Whisker plots describing HIC and RIC

Kinematic data is also presented relative to ball delivery distance (Table 3), and used to calculate HIC and RIC (Fig. 3). All three metrics increase with increasing pass distance, in line with increased PLA, PAA and PAV.

**Table 3.** Mean head kinematic data across the three pass distances

	*n*	Linear acceleration (g)	Angular acceleration (rad/s^2^)	Angular velocity (rad/s)
		Mean (SD)	Mean (SD)	Mean (SD)
Short pass	20	18.72 (3.73)	1242.30 (342.40)	5.50 (1.27)
Medium pass	21	26.57 (5.51)	1765.10 (369.90)	6.77 (1.69)
Long pass	24	31.90 (7.28)	2127.40 (663.95)	9.05 (1.75)

### Brain Strain Response Analysis

**Fig. 4:**
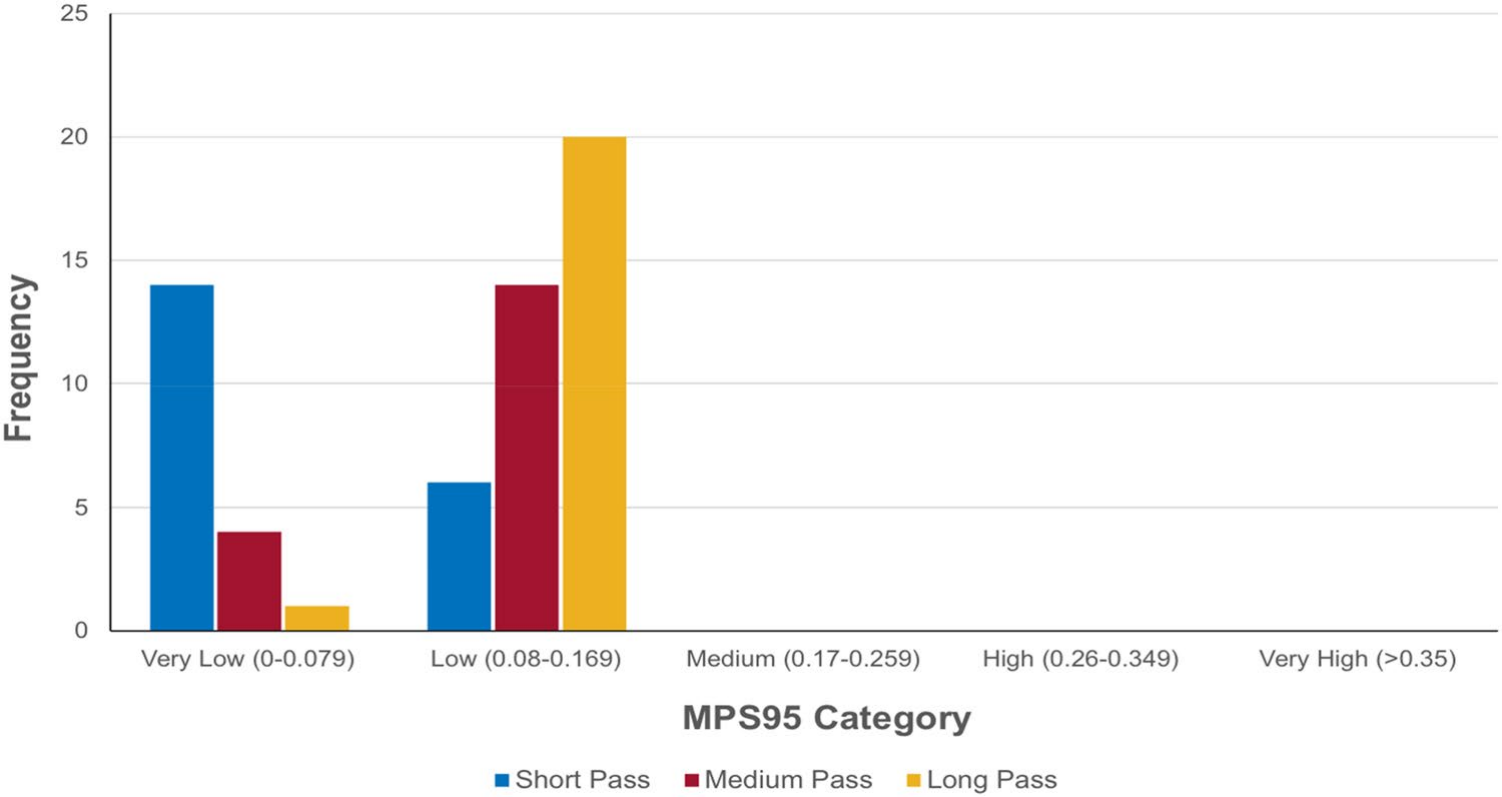
Frequency of 95th percentile MPS based on pass distance categorised by predicted brain injury risk level.

Kinematic data was inputted into the FE model to estimate the brain strain response across all headers. MPS and CSDM were outputted as strain-response metrics. Comparisons of the frequency of estimated brain strain for each pass distance can be found in Fig. 4. All recorded events presented MPS values categorised as very low or low, brain injury risk. The short pass distance produced a greater number of impacts that had a very low risk of brain injury, than low risk. The medium and long passes saw a greater number of MPS events in the low, than very low risk, category.

**Fig. 5:**
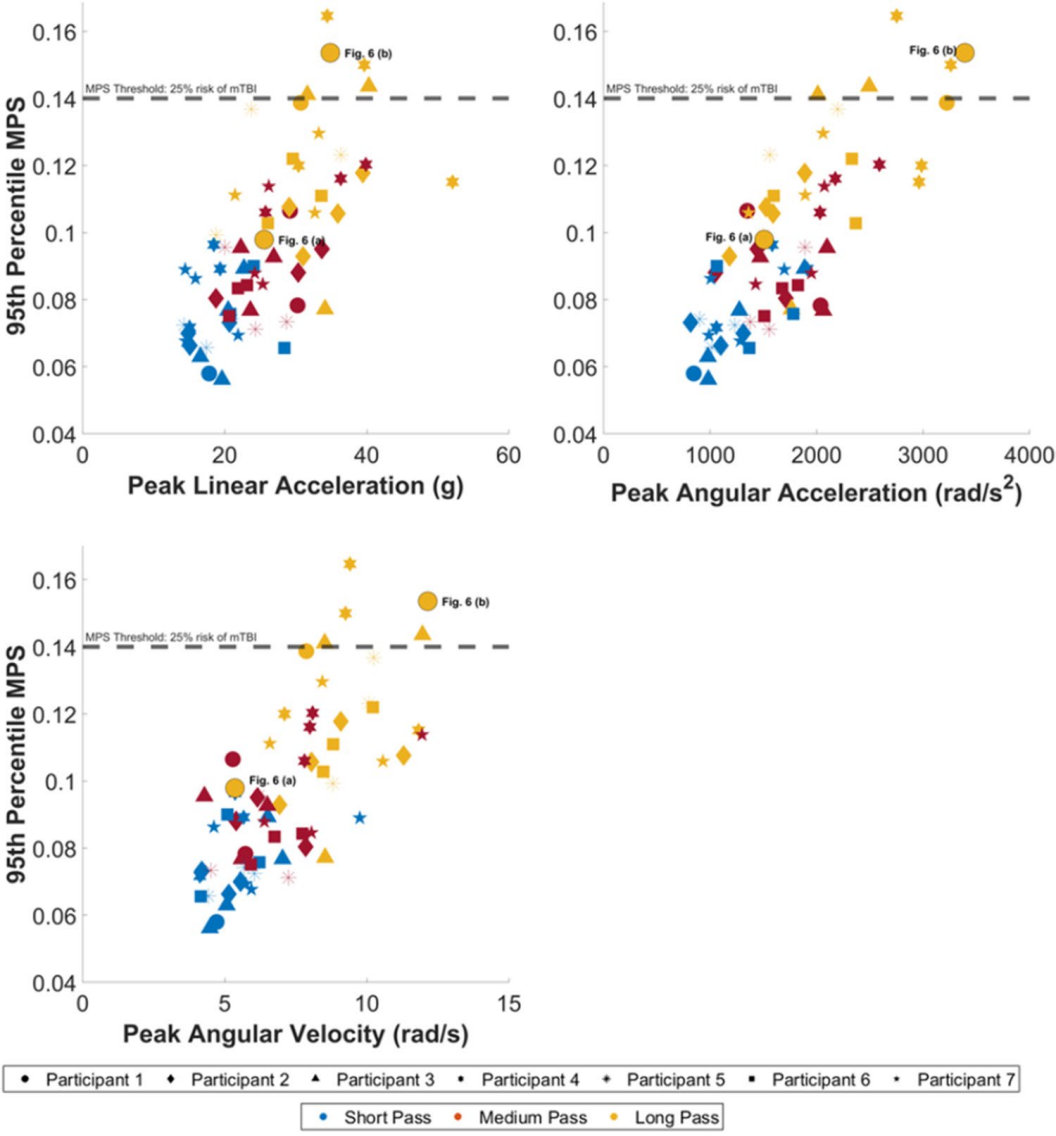
Kinematic data against MPS for 7 participants across three pass delivery distances

**Fig. 6:**
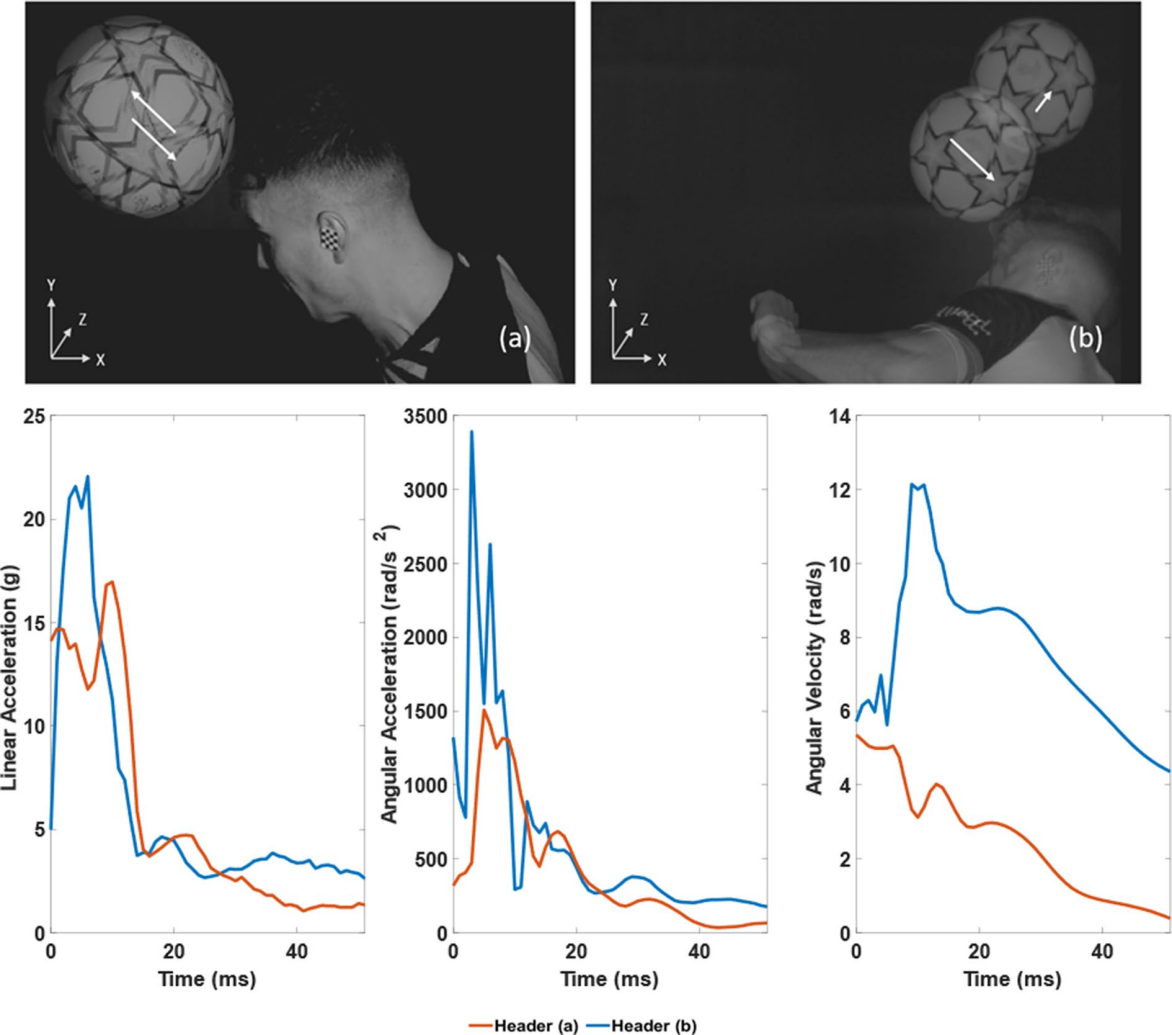
Examples of headers **a** returning the ball in the same direction, **b** deflecting the ball in a different trajectory and, **c** corresponding kinematic time-series data.

The relationship between kinematic measures and strain-based values were then investigated to further understand the brain response (Fig. 5). The expected positive correlation between pass distance and risk is evident in all plots, with 5 headers exceeding a 0.14 MPS threshold, which defines a 25% risk of mTBI [45]. Each participant experiences a similar kinematic and strain response when considering their individual heading events, at different pass distances. Large variations do exist when considering the relative performance of individuals across the different heading distances. High-speed video analysis was investigated to better understand this variation. Fig. 6. presents two headers from the same player at the same distance. Fig. 6a shows the player returning the ball along the pathway of the inbound direction, whilst Fig. 6b demonstrates poor technique as the player deflects the ball onto a different trajectory. The kinematic metrics and resultant MPS value for the two headers are presented in Fig. 5.

**Fig. 7:**
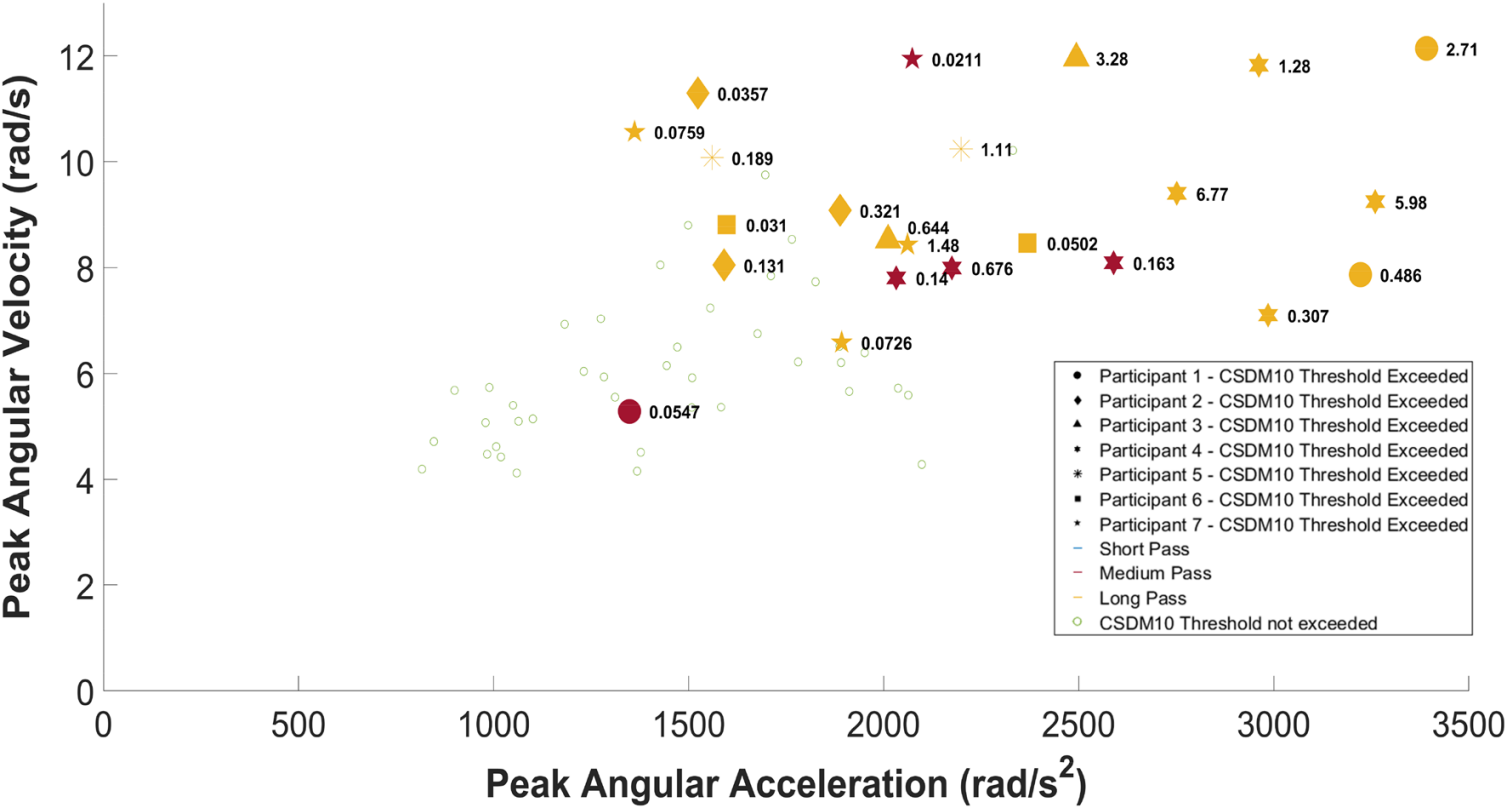
CSDM values against PAV and PAA for seven participants across three distances

CSDM10 values were investigated against PAA and PAV (Fig. 7). CSDM10 values for each participant are shown against different pass distances, though only medium and long pass distances resulted in MPS values exceeding a 0.1 threshold. Of the total 65 headers, 20 exceeded an MPS threshold of 0.1 (CSDM10), five from a medium and 15 from a long pass distance.

##  Discussion

This study investigated the effect of heading on brain strain, given the increasing concern that repeated impacts can lead to long-term neurodegenerative diseases. A homogenous cohort of amateur male footballers was chosen to minimise participant variability, thus better accomplishing the primary aim of the study. Increasing pass distance was generally associated with higher head accelerations; however, none of the 65 headers were considered injurious when compared to HIC and RIC thresholds. It should be recalled, though, that while such metrics are used widely to describe injury risk in sport, these thresholds are derived from higher energy applications and so concussive injuries, being too insensitive for the low magnitude impacts associated with soccer.

FE modelling of tissue strain provides opportunity for a more sensitive, if in-direct, prediction of brain injury. A positive trend was observed when plotting kinematics against brain strain, which is consistent with the results of other studies [22, 53–58]. A CSDM10 injury risk threshold of 0.107 (10.7%)—derived from [59] to represent where 10% of all elements have exceeded a critical strain associated with 25% risk of mTBI, was not breached. Applying a 0.14 MPS threshold resulted in 5 headers from the longest distance being associated with a 25% mTBI risk. As 23 headers were recorded from this distance, approximately 1-in-5 breached this injury threshold. Should this be translatable to game play, then these data suggest heading should be considered a concussion risk [60–64]. Indeed, this ratio will likely be greater when including ‘higher force’ headers, from distances > 35 m, which is the current threshold for the Football Association’s practice restrictions.

Assuming that one header was concussive in this study, then 64 (or 98%) are sub-concussive, though importantly they still likely pose an injury risk based on accumulated trauma, especially when multiplied through a playing season and career. Sub-concussive severity thresholds have yet to be established due to a lack of associated clinical sequalae, though magnetic resonance-based metrics are being explored [65]. FE modelling was used to estimate brain strain in youth American football impacts for non-concussed players. Though using a different FE model, namely the atlas-based brain model, Miller et al. [66–68] reported neurostructural changes corresponding to ninety-fifth percentile MPS values, which were similar to the higher values reported here (0.1525). It may be, therefore, that the cumulative effect of heading long-distance passes is comparable to those neurological outcomes associated with repeated impacts in American youth football players.

Significant intra-participant variation was observed in the kinematic and strain-based metrics. High speed video was utilised for further investigation. Fig. 6 a. demonstrates sound technique, heading the ball back in the direction of travel. The same participant then poorly directed another long pass (Fig. 6b). This has, however, provided an inadvertent insight into how rotation-inducing headers can increase head injury risk (Fig. 7) with poor technique breaching the 0.14 MPS value (25% risk of concussion). This is consistent with studies across other sports that have explored links between rotation-inducing impacts and injury risk factors [69–72]. Wililko et al. reported a 68% risk of concussion due to rotational accelerations from punches delivered from Olympic boxers [70], whilst others reported a ‘hook’ boxing punch to induce significantly greater rotational acceleration compared to other punch types, resulting in a 96% risk of concussion. Interestingly, punches resulting in higher rotational accelerations also increased the likelihood of injury from linear acceleration, a 23% increase in concussion risk compared to the second most injurious punch [72]. Many studies have reported rotational acceleration to have a greater influence on strain values, though linear acceleration still creates injury risk, particularly when considering concussions and other mTBI [73, 74].

Demonstrating the validity of our data is challenging, given the variation in data across published studies considering head/brain kinematics in soccer heading. Saunders et al. used skin patch sensors to capture on-field PLA and PAA values in collegiate-level males. Both training and gameplay PLA values were lower than this study (17.26 g and 21.92 g, respectively), however PAA values were much higher (2704.2 rad/s^2^ and 3825 rad/s^2^) [53]. Their use of skin-mounted accelerometers may have contributed to their significantly higher values, with Wu et al. [16] noting the frequent inclusion of artefactual data caused by soft tissue deformation. Our approach, using iMGs, provides a more rigid coupling between the sensors and skull. Another study used a custom tight-fitting elastic cap to measure PLA and PAA in a mixed cohort of youth, high school, and collegiate players. Results for men were reported as 27.6 g and 2219 rad/s^2^ for PLA and PAA, respectively. These were similar to results in this study (26 g and 1730 rad/s^2^); however, the collegiate level mixed cohort results were 30% and 47% greater for PLA and PAA, respectively (34.8 g and 2792 rad/s^2^) [55]. The inclusion of women in the collegiate cohort is expected to increase peak values due to women recording higher kinematic data [53, 55, 57]. Sokol-Randell et al. used iMGs to capture PLA, PAA and PAV in a cohort of ten university level males during gameplay across a season. Three similar pass distances reported kinematic values approximately 50% less than our values [58]. Miller et al. used iMGs and 3 similar pass distances during on-field sessions, with PLA, PAV and PAA values varying between 4 and 42% lower than the data in this study [22]. This was recorded in youth female soccer (under 14 years old) while we reported 21-year-old males, meaning that the variation in values is unsurprising. Our data may be higher than that recorded in-game, due to the athlete being focussed exclusively on performing against the objective, as has been reported elsewhere [75]. Another study used iMGs and FE modelling to quantify head kinematic data and estimate brain strain response. A pass distance between the short and medium used in this study, resulted in smaller PLA, PAA and PAV values by 23%, 25% and 9%, respectively, while MPS values were 55% smaller. Lower values reported are likely due to the study considering adolescent players [76].

The results of this study provide a greater understanding of the head kinematics and brain strain generated during a full-scale, laboratory investigation. These values indicate that heading a soccer ball appears almost exclusively to achieve sub-concussive impacts, though these are still associated with brain tissue strains that breach recognised injury thresholds. Given this, plus how these headers are not considered ‘high force’, and how soccer encourages repetitive heading, further investigation is warranted to better understand the potential long-term effects of ball-head impacts, exploring how these risks can be managed to the benefit of the players and the game. Future studies should predominately focus on the effect of header configurations frequently seen in game play scenarios on brain injury risk, including expansion of the cohort to better represent female and younger players.

### Supplementary Information

Below is the link to the electronic supplementary material.

Supplementary file1 (PDF 305 KB)

## References

[1] FIFA Communications Division. FIFA Big Count 2006. Information Services. 2007. https://digitalhub.fifa.com/m/4b896d21ec47be02/original/vrnjcgakvf7nds6sl5rx-pdf.pdf. Accessed June 2023.

[2] Krustrup P, Aagaard P, Nybo L, Petersen J, Mohr M, Bangsbo J (2010). Recreational football as a health promoting activity: a topical review. Scand. J. Med. Sci. Sports.

[3] Tenforde AS, Fredericson M (2011). Influence of sports participation on bone health in the young athlete: a review of the literature. PM&R.

[4] Cantu, R., C. Nowinski, C. R.-E. IoSLa, ed. Santa, and undefined 2015, Safer Soccer White Paper The Neurological Consequences of Heading in Soccer *chc.edu*. 10.1001/jamapediatrics.2013.4518.

[5] Stephens R, Rutherford A, Potter D, Fernie G (2007). “Neuropsychological impairment as a consequence of football (soccer) play and football heading: a preliminary analysis and report on school students (13–16 years). Child Neuropsychol..

[6] New heading guidance for English football during 2021–2022 season. Accessed 26 Jun 2023. Available: https://www.thefa.com/news/2021/jul/28/20210728-new-heading-guidance-published

[7] Martland HS (1928). PUNCH DRUNK. J. Am. Med. Assoc..

[8] Lee EB, Kinch K, Johnson VE, Trojanowski JQ, Smith DH, Stewart W (2019). Chronic traumatic encephalopathy is a common co-morbidity, but less frequent primary dementia in former soccer and rugby players. Acta Neuropathol.

[9] Solomon GS, Zuckerman SL (2015). Chronic traumatic encephalopathy in professional sports: Retrospective and prospective views. Brain Inj.

[10] McKee AC (2009). Chronic traumatic encephalopathy in athletes: progressive tauopathy after repetitive head injury. J Neuropathol. Exp. Neurol..

[11] Mackay DF, Russell ER, Stewart K, MacLean JA, Pell JP, Stewart W (2019). Neurodegenerative disease mortality among former professional soccer players. N. Engl. J. Med..

[12] McKee AC, Alosco ML, Huber BR (2016). Repetitive head impacts and chronic traumatic encephalopathy. Neurosurg. Clin. N. Am..

[13] Russell ER, MacKay DF, Stewart K, MacLean JA, Pell JP, Stewart W (2021). Association of field position and career length with risk of neurodegenerative disease in male former professional soccer players. JAMA Neurol..

[14] Liu Y (2020). Validation and comparison of instrumented mouthguards for measuring head kinematics and assessing brain deformation in football impacts. Ann. Biomed. Eng..

[15] Camarillo DB, Shull PB, Mattson J, Shultz R, Garza D (2013). An instrumented mouthguard for measuring linear and angular head impact kinematics in American football. Ann. Biomed. Eng..

[16] Wu LC (2016). In vivo evaluation of wearable head impact sensors. Ann. Biomed. Eng..

[17] Clark, J. M., A. Post, T. B. Hoshizaki, and M. D. Gilchrist: Determining the relationship between linear and rotational acceleration and MPS for different magnitudes of classified brain injury risk in ice hockey. International Research Council on the Biomechanics of Injury (IRCOBI), 2015.

[18] Liu Y (2021). Time window of head impact kinematics measurement for calculation of brain strain and strain rate in american football. Ann. Biomed. Eng..

[19] Levy Y, Bian K, Patterson L, Ouckama R, Mao H (2021). Head kinematics and injury metrics for laboratory hockey-relevant head impact experiments. Ann. Biomed. Eng..

[20] Cecchi NJ (2021). Identifying factors associated with head impact kinematics and brain strain in high school american football via instrumented mouthguards. Ann. Biomed. Eng..

[21] Press JN, Rowson S (2017). Quantifying head impact exposure in collegiate women’s soccer. Clin. J. Sport Med..

[22] Miller LE (2020). Characterizing head impact exposure in youth female soccer with a custom-instrumented mouthpiece. Res. Sports Med..

[23] Hanlon EM, Bir CA (2012). “Real-time head acceleration measurement in girls' youth soccer. Med. Sci. Sports Exerc..

[24] Gysland SM, Mihalik JP, Register-Mihalik JK, Trulock SC, Shields EW, Guskiewicz KM (2012). The relationship between subconcussive impacts and concussion history on clinical measures of neurologic function in collegiate football players. Ann. Biomed. Eng..

[25] Rowson S (2014). Can helmet design reduce the risk of concussion in football?. J. Neurosurg..

[26] Duhaime A-C (2012). Spectrum of acute clinical characteristics of diagnosed concussions in college athletes wearing instrumented helmets. J. Neurosurg..

[27] O'Connor KL, Rowson S, Duma SM, Broglio SP (2017). Head-impact-measurement devices: a systematic review. J. Athletic Train..

[28] Jones CM, Rowan M (2022). Brown, “validation of an instrumented mouthguard”. Sensors.

[29] FIFA: FIFA Quality Programme for Footballs (outdoor, futs al and beach soccer footballs. Testing Manual. *FIFA Quality Programme*, 2018.

[30] Jones CM, Austin K, Augustus SN, Nicholas KJ, Yu X, Baker C, Chan EYK, Loosemore M, Ghajari M (2023). An instrumented mouthguard for real-time measurement of head kinematics under a large range of sport specific accelerations. Sensors (Basel).

[31] Horgan TJ, Gilchrist MD (2010). “Influence of FE model variability in predicting brain motion and intracranial pressure changes in head impact simulations. Int. J. Crashworthines.

[32] Horgan TJ, Gilchrist MD (2003). The creation of three-dimensional finite element models for simulating head impact biomechanics. Int. J. Crashworth..

[33] Zhou C, Khalil TB, King AI (1995). A new model comparing impact responses of the homogeneous and inhomogeneous human brain. SAE Trans.

[34] Zhang, K et al.: Recent advances in brain injury research: a new human head model development and validation. SAE Technical Papers, 2001, doi: 10.4271/2001-22-0017. 10.4271/2001-22-0017 17458754

[35] Willinger R, Taleb L, Kopp CM (1995). Modal and temporal analysis of head mathematical models. J. Neurotrauma.

[36] Ruan, J: Impact biomechanics of head injury by mathematical modeling. 1994. Accessed 21 June 2023 https://search.proquest.com/openview/10b0beacdf11cef9e3d02bc30c4a0e45/1?pq-origsite=gscholar&cbl=18750&diss=y.

[37] Kleiven, S., von Humboldt H.-P.: Consequences of brain size following impact in prediction of subdural hematoma evaluated with numerical techniques. *ircobi.org*, 2001, Accessed 21 June 2023. http://www.ircobi.org/wordpress/downloads/irc0111/2001/Session3/3.1.pdf

[38] Post A, Hashim E, Hoshizaki TB, Gilchrist MD, Cusimano MD (2021). A preliminary examination of the relationship between biomechanical measures and structural changes in the brain. Trauma (UK).

[39] Oeur RA (2015). A comparison of head dynamic response and brain tissue stress and strain using accident reconstructions for concussion, concussion with persistent postconcussive symptoms, and subdural hematoma. J. Neurosurg..

[40] Nahum AM, Smith R, Ward CC (1977). Intracranial pressure dynamics during head impact. SAE Technical Papers.

[41] Hardy WN, Foster CD, Mason MJ, Yang KH, King AI, Tashman S (2001). Investigation of head injury mechanisms using neutral density technology and high-speed biplanar X-ray. Stapp. Car. Crash J..

[42] Doorly MC, Gilchrist MD (2007). The use of accident reconstruction for the analysis of traumatic brain injury due to head impacts arising from falls. Comput. Methods Biomech. Biomed. Eng..

[43] Post A, Walsh ES, Hoshizaki TB, Gilchrist MD (2012). Analysis of loading curve characteristics on the production of brain deformation metrics. Proc. Inst. Mech. Eng..

[44] Adams R, Soe S, Theobald P (2023). Optimisation of an elastomeric pre-buckled honeycomb helmet liner for advanced impact mitigation. Smart Mater. Strct..

[45] R. A. Oeur, Michael D. Gilchrist & T. Blaine Hoshizaki, “Parametric study of impact parameters on peak head acceleration and strain for collision impacts in sport”, *International Journal of Crashworthiness*, 26(1), pp. 16-25, 2021, Doi: 10.1080/13588265.2019.1634336

[46] Clark JM, Post A, Hoshizaki TB, Gilchrist MD (2016). Protective capacity of ice hockey helmets against different impact events. Ann Biomed Eng.

[47] Clark JM, Hoshizaki TB, Gilchrist MD (2020). Event-specific impact test protocol for ice hockey goaltender masks. Sports Biomech..

[48] Michio Clark J, Post A, Blaine Hoshizaki T, Gilchrist MD (2018). Distribution of brain strain in the cerebrum for laboratory impacts to ice hockey goaltender masks. ASME. J Biomech Eng.

[49] Hassan MH, Mohd Anni MA, Tan FY, Johari NH, Omar MN (2022). The influence of ball impact angle on the brain deformation in soccer heading: a finite element analysis. Hum.-Cent. Technol. Better Tomorrow.

[50] Kleiven S (2007). Predictors for traumatic brain injuries evaluated through accident reconstructions. Stapp Car. Crash J..

[51] Kimpara H, Iwamoto M (2012). Mild traumatic brain injury predictors based on angular accelerations during impacts. Ann. Biomed. Eng..

[52] Giordano C, Kleiven S (2014). Evaluation of axonal strain as a predictor for mild traumatic brain injuries using finite element modeling. Stapp Car. Crash J..

[53] Saunders TD, Le RK, Breedlove KM, Bradney DA, Bowman TG (2020). Sex differences in mechanisms of head impacts in collegiate soccer athletes. Clin. Biomech..

[54] Filben TM, Pritchard NS, Hanes-Romano KE, Miller LE, Miles CM, Urban JE, Stitzel JD (2021). Comparison of women's collegiate soccer header kinematics by play state, intent, and outcome. J. Biomech..

[55] Caccese JB (2017). Head and neck size and neck strength predict linear and rotational acceleration during purposeful soccer heading. Taylor Francis.

[56] Harriss A, Johnson AM, Walton DM, Dickey JP (2019). Head impact magnitudes that occur from purposeful soccer heading depend on the game scenario and head impact location. Musculoskelet. Sci. Pract..

[57] Tierney RT, Higgins M, Caswell SV, Brady J, McHardy K, Driban JB, Darvish K (2008). Sex differences in head acceleration during heading while wearing soccer headgear. J. Athl. Train.

[58] Sokol-Randell D, Stelzer-Hiller OW, Allan D, Tierney G (2023). Heads Up! A biomechanical pilot investigation of soccer heading using instrumented mouthguards (iMGs). Appl. Sci..

[59] Giordano C, Kleiven S (2014). Evaluation of axonal strain as a predictor for mild traumatic brain injuries using finite element modeling. Stapp. Car. Crash J..

[60] McKee AC (2009). Chronic traumatic encephalopathy in athletes: progressive tauopathy after repetitive head injury. J. Neuropathol. Exp. Neurol..

[61] Gavett BE, Stern RA, McKee AC (2011). Chronic traumatic encephalopathy: a potential late effect of sport-related concussive and subconcussive head trauma. Clin. Sports Med..

[62] Erlanger DM, Kutner KC, Barth JT, Barnes R (1999). Neuropsychology of sports-related head injury: dementia Pugilistica to post concussion syndrome. Clin. Neuropsychol..

[63] Baugh CM (2012). Chronic traumatic encephalopathy: neurodegeneration following repetitive concussive and subconcussive brain trauma. Brain Imaging Behav..

[64] Bailes JE, Petraglia AL, Omalu BI, Nauman E, Talavage T (2013). Role of subconcussion in repetitive mild traumatic brain injury. J. Neurosurg..

[65] Martini D, Eckner J, Kutcher J, Broglio SP (2013). Subconcussive head impact biomechanics: comparing differing offensive schemes. Med. Sci. Sports Exerc..

[66] Miller LE (2021). Brain strain: computational model-based metrics for head impact exposure and injury correlation. Ann. Biomed. Eng..

[67] Miller LE (2022). Cumulative strain-based metrics for predicting subconcussive head impact exposure–related imaging changes in a cohort of American youth football players. J. Neurosurg. Pediatr..

[68] Davenport EM (2014). Abnormal white matter integrity related to head impact exposure in a season of high school varsity football. J. Neurotrauma.

[69] Karton C, Hoshizaki TB (2018). Concussive and subconcussive brain trauma: the complexity of impact biomechanics and injury risk in contact sport. Handbook Clin. Neurol..

[70] Walilko TJ, Viano DC, Bir CA (2005). Biomechanics of the head for Olympic boxer punches to the face. Br. J. Sports Med..

[71] Tucker R (2017). Risk factors for head injury events in professional rugby union: a video analysis of 464 head injury events to inform proposed injury prevention strategies. Br. J. Sports Med..

[72] Viano D, Casson I, Pellman E (2005). Concussion in professional football: comparison with boxing head impacts—part 10. Neurosurgery.

[73] Wright R, Post A, Hoshizaki TB, Ramesh KT (2012). A multiscale computational approach to estimating axonal damage under inertial loading of the head. J. Neurotrauma.

[74] Post A, Hoshizaki TB, Gilchrist M, Cusimano M (2017). Peak linear and rotational acceleration magnitude and duration effects on maximum principal strain in the corpus callosum for sport impacts. J. Biomech..

[75] Newman JA, Beusenberg MC, Shewchenko N, Withnall C, Fournier E (2005). Verification of biomechanical methods employed in a comprehensive study of mild traumatic brain injury and the effectiveness of American football helmets. J. Biomech..

[76] Huber CM, Patton DA, Maheshwari J, Zhou Z, Kleiven S, Arbogast KB (2023). Finite element brain deformation in adolescent soccer heading. Comput. Methods Biomech. Biomed. Eng..

