# Supplementary material for: Investigation of Head Kinematics and Brain Strain Response During Soccer Heading Using a Custom-Fit Instrumented Mouthguard
Source: Ann Biomed Eng. 2024 Jan 19;52(4):934–45. doi: 10.1007/s10439-023-03430-8 (PMC10940496; doi:10.1007/s10439-023-03430-8)
Supplement: Supplementary file 1 — Supplementary file1 (PDF 305 KB) [file 10439_2023_3430_MOESM1_ESM.pdf]

Investigation of Head Kinematics and Brain Response During Soccer Heading Using a Custom-Fit Instrumented Mouthguard

M. Barnes-Wood[1,2], H. McCloskey[1,2], S. Connelly[3], M.D. Gilchrist[4], A. Ni Annaidh[4], and P. Theobald[1]

1 Cardiff School of Engineering, Cardiff University, The Parade, Cardiff, CF24 3AA, UK

2 Charles Owen & Co, Croesfoel Industrial Park, Wrexham, LL14 4BJ, UK.

3 Football Association of Wales (*FIFA Medical Centre of Excellence)*, Hensol, Pontyclun, CF72 8JY, UK.

4 School of Mechanical and Materials Engineering, University College Dublin, Belfield, Dublin 4, Ireland.

Corresponding author: Peter Theobald

Corresponding author email: TheobaldPS@cardiff.ac.uk

Supplementary Information 1

The coordinate system for the iMG can be seen in Fig. 1. For complete comparison of acceleration data, the iMG outputs were translated to match the sign convention of the FE head model where (+X points forward with respect to the UCDBTM; +Y points right; +Z points downward). The sign convention changes are detailed in Table 1.

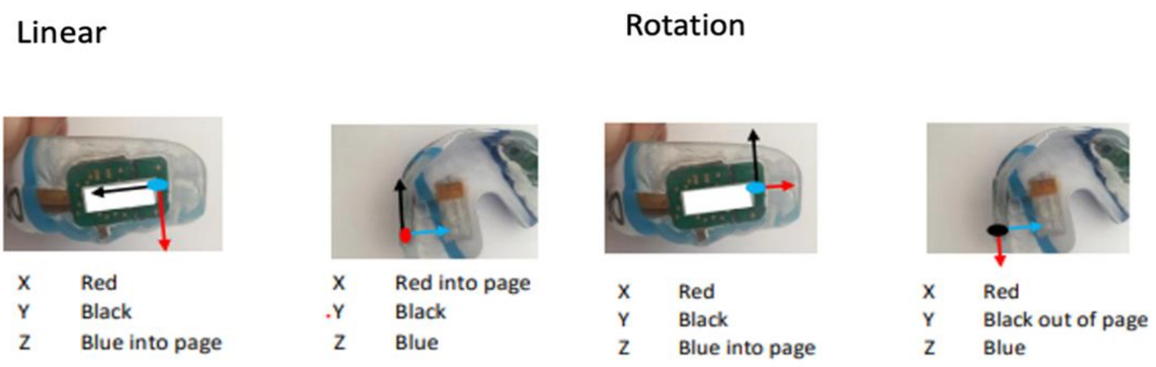

***Fig 1**. Orientation of linear and rotational accelerometer sensors*

***Table 1**. Coordinate convention of iMG sensor and FE Model for linear and angular acceleration*

Linear Acceleration		Angular Acceleration	
iMG sensor	FE head model	iMG sensor	FE head model
X	Z	X	-X
Y	X	Y	-Z
Z	Y	Z	Y

Sagittal, coronal and axial- plane MRI data were used to transform the measured iMG data to estimate the accelerations at the CoG of the FE head model by accounting for angular offset within the mouth. A dummy mouthguard with a 3D printed sensor board from TPE 45 Shore A material with 50% infill to achieve suitable contrast between the sensors and participants dental features during MRI scanning. Rotational axes and offset angles were extracted using image processing techniques as described in Fig. 2 and related to the CoG of the head.

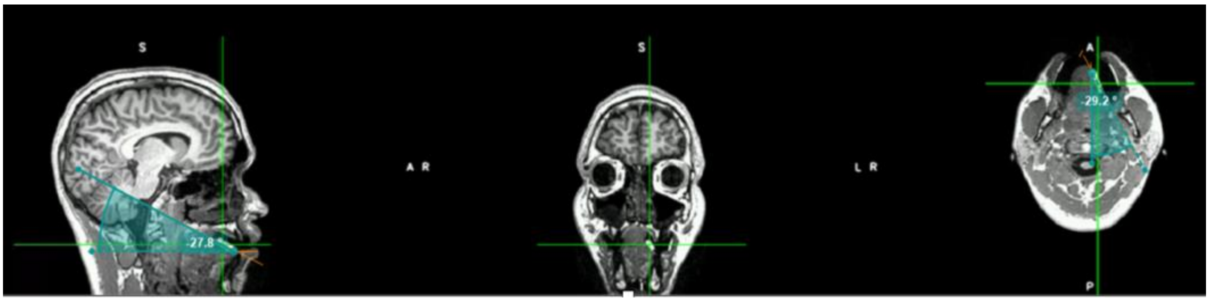

***Fig 2**. Sagittal, coronal and axial- plane MRI data to identify angle of offset of mouthguard sensors due to dental features*

Rotational offset about the Z-axis (-29.2°) for linear acceleration was transformed for X- and Y- directions using equations 1 and 2.

$$a_{x(z)} = [(a_{m(x)} \times \cos(-29.2)) + (a_{m(y)} \times \sin(-29.2))]$$

$$a_{y(z)} = [(-a_{m(x)} \times \sin(-29.2) + (a_{m(y)} \times \cos(-29.2))]$$

Where $a_{x(z)}$ and $a_{y(z)}$ is the acceleration in the x- and y-direction about Z, respectively, $a_{m(x)}$ is the raw translated iMG data in the x-direction and $a_{m(y)}$ is the raw translated iMG data in the y-direction.

Rotational offset about the X-axis(-27.8°) was transformed in Y- and Z- directions using equations 3 and 4.

$$a_{y(x)} = [(a_{m(y)} \times \cos(-27.8)) + (a_{m(z)} \times \sin(-27.8))]$$

$$a_{z(x)} = [(-a_{m(y)} \times \sin(-27.8) + (a_{m(z)} \times \cos(-27.8))]$$

Where $a_{y(x)}$ and $a_{z(x)}$ is the acceleration in the y- and z-direction about X, respectively, $a_{m(y)}$ is the raw translated iMG data in the y-direction and $a_{m(z)}$ is the raw translated iMG data in the z-direction.

Investigation of Head Kinematics and Brain Response During Soccer Heading Using a Custom-Fit Instrumented Mouthguard

M. Barnes-Wood[1,2], H. McCloskey[1,2], S. Connelly[3], M.D. Gilchrist[4], A. Ni Annaidh[4], and P. Theobald[1]

1 Cardiff School of Engineering, Cardiff University, The Parade, Cardiff, CF24 3AA, UK

2 Charles Owen & Co, Croesfoel Industrial Park, Wrexham, LL14 4BJ, UK.

3 Football Association of Wales (*FIFA Medical Centre of Excellence)*, Hensol, Pontyclun, CF72 8JY, UK.

4 School of Mechanical and Materials Engineering, University College Dublin, Belfield, Dublin 4, Ireland.

Corresponding author: Peter Theobald

Corresponding author email: TheobaldPS@cardiff.ac.uk

Supplementary Information 2

Research from Post et al. [43] suggests that the UCDBTM may generate large strains with only linear acceleration as an input. To ensure accuracy of the strain reported within this study, separate analysis was performed on a randomly selected simulation set.

Table 2. 95th Percentile MPS values for linear and rotational acceleration and linear rotational only inputs

			**95th Percentile Maximum Principal Strain**	
**Simulation No.**	**Participant No.**	**Header No.**	Linear + rotational acceleration	Linear acceleration only
1	1	9	0.1390	0.0121
2	2	1	0.066	0.0136
3	3	10	0.118	0.0200
4	4	5	0.096	0.0117
5	5	5	0.096	0.0155
6	6	7	0.099	0.0120
7	7	8	0.137	0.0104

Table 1 presents the result of this analysis which confirms that with linear acceleration as the only input to the model, large strains were not observed. Thus, the strains reported in this study are reported with confidence. Post et al. analysed linear acceleration inputs larger than those in this study which may be reflect their findings of large strains, perhaps suggesting the model is better suited for analyses of impacts resulting in lower linear acceleration values.
